# Ultrasound-Stimulated PVA Microbubbles as a Green and Handy Tool for the Cleaning of Cellulose-Based Materials

**DOI:** 10.3390/gels9070509

**Published:** 2023-06-23

**Authors:** Leonardo Severini, Alessia D’Andrea, Martina Redi, Sultan B. Dabagov, Valeria Guglielmotti, Dariush Hampai, Laura Micheli, Rocco Cancelliere, Fabio Domenici, Claudia Mazzuca, Gaio Paradossi, Antonio Palleschi

**Affiliations:** 1 Department of Chemical Science and Technologies, University of Rome “Tor Vergata”, Via Della Ricerca Scientifica 1, 00133 Rome, Italy; leonardo.severini@uniroma2.it (L.S.); alessia.dandrea@alumni.uniroma2.eu (A.D.); martina.redi@uniroma2.it (M.R.); laura.micheli@uniroma2.it (L.M.); rocco.cancelliere@uniroma2.it (R.C.); gaio.paradossi@uniroma2.it (G.P.); antonio.palleschi@uniroma2.it (A.P.); 2 INFN-LNF, XLab Frascati, Via Enrico Fermi 54, 00044 Rome, Italy; sultan.dabagov@lnf.infn.it (S.B.D.); valeria.guglielmotti@lnf.infn.it (V.G.); dariush.hampai@lnf.infn.it (D.H.); 3 RAS P.N. Lebedev Physical Institute, Leninsky pr 53, 119991 Moscow, Russia; 4 National Research Nuclear University MEPhI, Kashirskoe Sh. 31, 115409 Moscow, Russia

**Keywords:** gels, microbubbles, poly(vinyl alcohol), cultural heritage, artworks, cleaning

## Abstract

One of the main issues in the cultural heritage field of restoration chemistry is the identification of greener and more effective methods for the wet cleaning of paper artefacts, which serve as witnesses to human history and custodians of cultural values. In this context, we propose a biocompatible method to perform wet cleaning on paper based on the use of 1 MHz ultrasound in combination with water-dispersed polyvinyl alcohol microbubbles (PVAMBs), followed by dabbing with PVA-based hydrogel. This method can be applied to both old and new papers. FTIR spectroscopy, X-ray diffraction, HPLC analysis, pH measurements and tensile tests were performed on paper samples, to assess the efficacy of the cleaning system. According to the results, ultrasound-activated PVAMB application allows for an efficient interaction with rough and porous cellulose paper profiles, promoting the removal of cellulose degradation byproducts, while the following hydrogel dabbing treatment guarantees the removal of cleaning materials residues. Moreover, the results also pointed out that after the treatment no thermal or mechanical damages had affected the paper. In conclusion, the readability of these kinds of artifacts can be improved without causing an alteration of their structural properties, while mitigating the risk of ink diffusion.

## 1. Introduction

Paper restoration plays a fundamental role in the cultural heritage preservation field. This material has served as the most popular writing medium for transmitting and storing information since the first century A.D. and this trend continues today. Restoration and maintenance have been relegated to traditional good practices, perhaps without a strong scientific foundation. Unfortunately, paper samples inevitably degrade over time depending on several factors such as intrinsic composition (e.g., rags or wood pulps) and the presence of additives and adverse environmental conditions (e.g., pollution, radiation, temperature, and humidity) [1,2]; cellulose, paper’s main component, is prone to deterioration through hydrolysis and/or oxidation reactions [3]. Hydrolysis processes lead to fragile paper, worsening its mechanical properties due to cellulose depolymerization; oxidation paths induce, in the first instance, yellowing and a loss of readability of both the text and the artistic content of the artifact, because of spurious chromophore formation, which, in turn, leads to hydrolysis [4,5,6]. Among the restoration operations of paper products that are usually carried out, cleaning is undoubtedly an important as well as delicate operation. This step allows the removal of substances resulting from the degradation processes of the paper, slowing down the natural aging process of the material and improving its optical quality. If this process is carried out carelessly, there is a risk of removing or destroying the sample’s constituent parts in addition to poor cleaning. The majority of the time, paper material is cleaned in a wet manner, commonly in a water bath. [7]. Unfortunately, a water bath, although largely employed, does not allow the tuning of the amount of water absorbed by the paper, discouraging its use on very fragile paper [8,9,10]. Additionally, this type of treatment is problematic when just a localized, intensely concentrated intervention is needed, as frequently happens working on the conservation of modern artifacts. Different strategies have been employed to overcome this risk and the most promising one is the use of polymer-based hydrogels; they are able to release moderate amounts of water, in a controlled and gradual way, and absorb dirt and cellulose byproducts due to capillarity [11,12,13]. Moreover, if properly functionalized/loaded, they can be employed for the removal of specific additives such as adhesive, glue and greasy compounds [14,15]. Indeed, paper artworks often contain other materials than paper sheets such as glue, adhesive residues and clips, the aging of which aggravates paper degradation [14,15]. This means that a complete restoration treatment must involve the removal of these materials, which have very complex and different physical–chemical properties (i.e., hydrophilicity). Due to the significant compositional differences between modern and ancient paper, this situation is complicated, and different restoration techniques need to be used [2,16,17]. In particular, up to the end of the XVIII century, Western paper was made of rags sized occasionally with animal glue or starch [18,19]. On the other hand, since XIX-century paper is usually made of wood pulp so, in addition to cellulose fibers, it may also include lignin, the amount of which depends on the production process: mechanical wood pulp contains about one-third of lignin (w/w), while in chemical pulp it is very low, i.e., non-detectable using infrared spectroscopy experiments [19]. Modern paper also contains additives such as alum, chalk, resin, starch, and synthetic waterproofing polymers such as Alkenyl Succinic Anhydride (ASA), or Alkyl Ketene Dimer (AKD) [20]. Furthermore, the evolution of paper manufacturing systems has also led to the production of a material with a shorter cellulose fiber length, which influences the overall physical–mechanical qualities of the paper sheets. Indeed, ancient paper was produced from more valuable raw materials (i.e., linen, hemp or cotton rags) and subjected to less invasive manufacturing processes with respect to those for modern paper (i.e., wood pulp) [2,16]. A whole cleaning process involves several steps, each one requiring the use of specific treatments such as gels loaded with amphipathic polymers, enzymes, or nanostructured fluids [21,22,23]. It must be emphasized that hydrogels require prolonged contact times (about 1 h) to carry out a satisfactory cleaning process. This is because their effectiveness is related to the ability to closely interact with the support; a paper surface is rough and irregular, with pores of a micrometer range, and thus decreases hydrogel cleaning efficiency [24,25,26]. All these limitations have been overcome through the use of microsized systems (i.e., Gellan gum microgels and poly(vinyl alcohol) microbubbles) which are able to penetrate more easily inside cellulose fibers, due to their colloidal particle size, and clean the support in a shorter time and with higher efficiency compared to the results of cleaning procedures using the same material in their monolithic hydrogel form. The use of synthetic polymers to obtain hydrogels and microsized systems has been pointed out to overcome limitations imposed by the natural ones and/or to improve some of their useful features in the cultural heritage preservation field. Synthetic ones indeed enjoy long-lasting stability, higher reproducibility, and allow greater control over the final properties of the system. One of them is polyvinyl alcohol (PVA), which is able to form stable chemical hydrogels and microgels via a reaction with its oxidation byproduct, *telechelic* PVA (*tel*-PVA) [27]. It has been demonstrated that PVA-based hydrogels showed excellent compatibility with cellulose-based supports and good cleaning efficiency for both ancient and modern paper samples, while maintaining the limitations of hydrogel systems, as described [28,29]. During the last decade, research has focused on expanding the use of echogenic PVA microbubbles (PVAMBs), i.e., micrometer-sized spheres made of an air core stabilized by a hydrophilic cross-linked PVA shell, from biomedicine (e.g., molecular imaging and targeted therapy) to cultural heritage [30,31,32]. Among the main reasons to use cross-linked polymer shell microbubbles (MBs), there are their robustness, chemical versatility, and biocompatibility. Thanks to the PVA-based elastomeric shell formulation, these polymer PVAMBs undergoing megahertz and low-intensity acoustic stimuli exhibit an unusual match of stability and sensitivity in the echogenic response. Specifically, high-frequency and low-amplitude cavitation can be easily induced in PVAMBs using safe, non-resonant US stimuli, producing in turn, microstreaming jets in their aqueous dispersion medium. When the cavitating water-dispersed PVAMBs are interfaced on a biopolymer structure, local efficient water agitation and permeation can be expected without causing structural damage. This may also boost non-solvent exchange effects, which are crucial to perform the efficient wet cleaning treatment of artifacts. Eventually, the versatility of the surface engineering of this MBs formulation confers it adhesive features, for the selective targeting of specific areas on heterogeneous structures. Due to these peculiar properties, PVAMBs are herein the subject of investigation as a promising cleaning tool for paper artifacts. In a previous work of our group, PVAMBS have been proposed for the localized removal of aged polystyrene-based synthetic adhesive residues from modern paper samples, thus constituting an environmentally friendly alternative to the use of organic solvents, which are toxic for operators [31]. Indeed, according to D’Andrea et al., thanks to the responsive properties induced by US action and their micrometric size (and therefore dimensions comparable to those of paper pores), PVAMBs perform a micro-mechanical action leading to a removal process resulting in yellow and brittle aged adhesive residues, present on the sample, in about 2 min (Figure S1) [31]. The aim of this work is to overcome a fundamental drawback in the restoration field, which is the optimization of an ad hoc cleaning protocol according to each different sample’s composition and the possible presence of contaminants (see above). Indeed, as already discussed, adhesive residues could be removed by means of net organic solvents, complex fluid media, or PVAMBS [17,31,33], while the cleaning of an aged paper sample, without specific contaminants, could be achieved using Gellan gum, poly(vinyl pyrrolidone) chains embedded in a poly(2-hydroxyethyl methacrylate) and/or PVA based hydrogel systems [13,15,29,34]. This means that a satisfying cleaning treatment consists of several successive steps. In this article, we investigate the possibility of using PVAMBs stimulated by US also to perform effective cleaning on modern and ancient paper to remove cellulose degradation hydrophilic byproducts from them, thus providing a unique and single-step cleaning process applicable to paper with different compositions and pollutants. Moreover, this strategy results time and cost savings (see “economic feasibility evaluation of PVAMBs system” paragraph in the Supplementary Materials). If assessed, this is an important feature as it means that the treatment can be not necessarily limited to zones containing adhesive residues. A great difference in the paper composition between ancient and modern paper (see above) exists, and a relevant amount of literature concerns cleaning procedures for ancient paper. Only recently, articles dealing with the cleaning of modern paper artworks appeared, highlighting the difficulty to apply the cleaning procedure for ancient paper to modern paper [25,28]. In this context, our idea is to propose a new “green” and sustainable tool for the simultaneous removal of hydrophilic (cellulose byproducts) and hydrophobic (i.e., aged adhesive) compounds, in a very short time, for both modern and ancient papers. Indeed, the presence of hydroxyl groups on PVA chains favors the establishment of weak dipolar interactions with detached cellulose degradation byproducts and hydrophobic residues (carrying carbonyl and carboxylic moieties able to interact with the PVA chains constituting the microbubble shell). A characterization of PVAMBs has been performed via acoustic attenuation spectroscopy, AFM spectroscopy and FTIR spectroscopy (see Section 4) as well as confocal laser scanning microscopy (CLSM) and dynamic light scattering (DLS). The last two techniques allowed us to determine the diameter distribution of the PVAMBs. In fact, to achieve an optimal interaction with paper, e PVAMBs size should be comparable with paper’s roughness. Due to the substantial differences between ancient and modern paper, the frequency (in the MHz frequency range), intensity and duty time of the applied US exploited in the proposed treatment were investigated. The aim was first to induce PVAMB cavitation, producing hydrodynamic flows [35], a condition necessary to avoid the violent and sudden breaking of the shell of the entire population of irradiated MBs, enabling the exploitation of PVAMB cavitation along the time of treatment. Secondly, this investigation was important to avoid invasive non-reversible paper damage (mechanical and thermal side effects were accurately checked). In the case of ancient paper, preliminary tests, regarding the tuning of parameters of the proposed protocol, were conducted on Whatman filter paper, as a reference, the composition and features of which resemble those typical of ancient paper (less manipulation and fewer chemical additives in the paper pulp; fewer coatings and a surface more permeable to water; more sensitivity to mechanical stress). After establishing the safety of the treatment on reference paper, the tool was then tested on ancient paper samples, hereafter called *Breviarium*, for the removal of cellulose degradation byproducts. To assess our idea, we deeply characterized modern and ancient paper samples, before and after PVAMB application, in order to evaluate their cleaning efficacy. Several experimental analyses were performed, such as attenuated total-reflectance Fourier transform infrared (ATR-FTIR) measurements, X-ray diffraction (XRD), colorimetric as well as pH measures, HPLC analysis and tensile tests.

## 2. Results and Discussion

### 2.1. Cleaning of Modern Paper Samples

**Figure 1:**
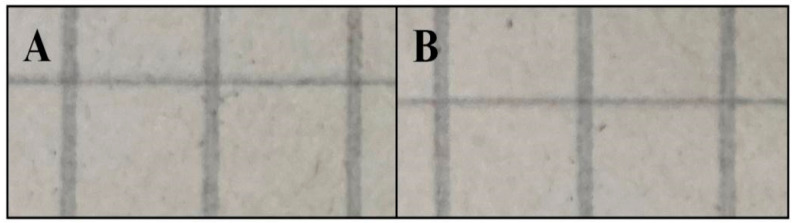
Optical images (obtained using visible light) of the modern paper sample before (**A**) and after treatment (**B**).

As previously discussed, a cleaning protocol based on PVAMBs stimulated using the US protocol has been applied only on adhesive residues in modern paper samples (Figure S1), but its effects have never been studied on modern and ancient paper themselves. First, we assessed the cleaning efficacy of PVAMBs on modern paper. In this case, the same protocol used for polystyrene adhesive removal (a duty cycle of 100%, intensity of 5 W/cm^2^ and frequency of 1 MHz) was applied. The proposed protocol can be so schematized as follows: the application of a small of PVAMB to be dispersed on the selected area; the setting of the sonicator with its own parameters characterizing the protocol; the application of the tip of the instrument (see Section 4) on the paper and performing the treatment for the desired time; the dabbing of the treated area with a piece of PVA chemical hydrogel to remove possible residuals and/or fragments of PVAMBs. Microscope images, reported in Figure 1, allow us to conclude that PVAMB treatment did not induce rips and ink fading, thus confirming its goodness. 

**Figure 2:**
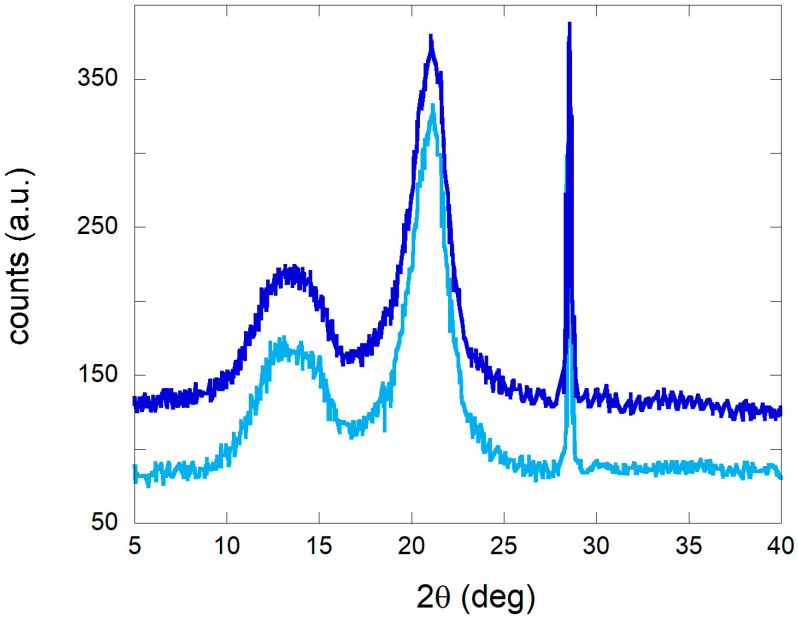
Diffraction pattern of notebook paper samples before (blue) and after (light blue) treatment.

Tensile test data show the preservation of the mechanical properties of the paper samples after performing cleaning with PVAMBs and US, indicating that treatment did not damage the paper. Stress and strain at break values obtained on cleaned and not cleaned samples are indeed similar, as they are 14 ± 2 MPa and (3.0 ± 0.5%), respectively, regardless of if they are treated or not. This is a very important finding, because in the first instance, the use of US on a cellulose-based artwork scan be avoided by considering that US can be employed to depolymerize cellulose [36,37]. However, the protocols reported in this work and those used for degrading cellulose are very different in US power, frequency and time. To describe this in more detail, the stable cavitation effects of PVAMBs are substantially below their resonance frequency and are sufficient to clean paper surfaces, thus resulting in their small oscillations, and hence avoiding cracking induced by US [38], excessive microstreaming flows and cavitation hotspots, and therefore, cellulose degradation [36,37]. This result was corroborated via XRD analysis. The diffraction pattern of untreated and treated notebook paper samples, reported in Figure 2, displays the presence of the same peaks attributable to the structure of cellulose I type β (JCPDS database—PDF card no. 3–289). Moreover, the first broadened peak, in the 10–25° range of the diffraction pattern, is assignable to the (10$\overset{-}{1}$) plane while the second one, which is more intense, is attributed to the (00$\overset{-}{2}$) plane, and is due to the crystalline part of the cellulose [39,40].

**Figure 3:**
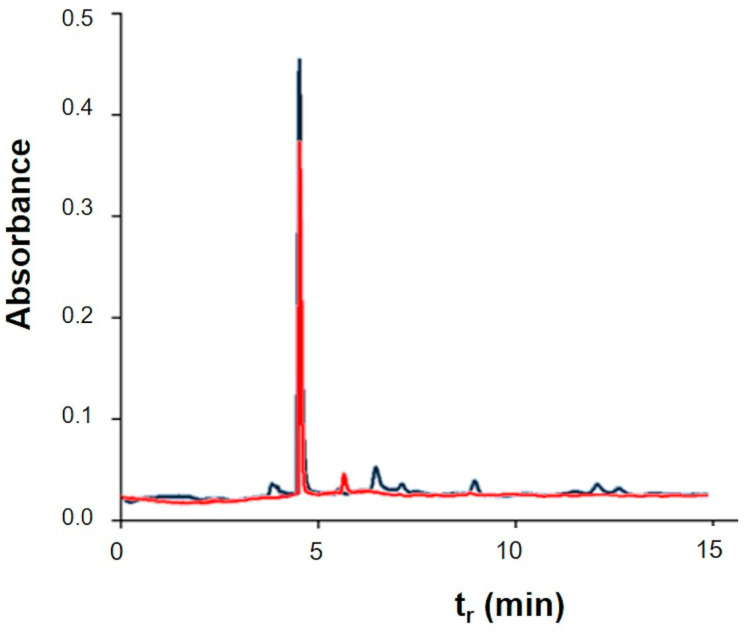
Chromatograms of aqueous extracts of notebook paper sample before (blue) and after (red) PVAMB cleaning.

According to Segal and colleagues’ methods (see Materials and Methods), these data can be used to calculate the crystallinity index (CI) of paper samples both before and after cleaning. CI values were stable at 0.8, before and after treatment, highlighting that the cleaning protocol did not damage the support. After assessing the absence of damage on the paper due to treatment, the effectiveness in cleaning was demonstrated by the pH values, HPLC, FTIR, and colorimetry experiments. As explained before, degradation causes indeed, a decrease in pH due to the formation of acid molecules from cellulose [41]. Because of cleaning, there was an increase in pH values ranging from 6.7 ± 0.1 to 7.2 ± 0.2, thus indicating that the treatment with MBs was able to remove acidic degradation products. With the aim of obtaining more insight into the removal of acid byproducts due to PVAMB treatment, HPLC analyses on water extracts of treated and non-treated paper fragments were performed. Using this technique, it is possible indeed, determine which kind of carboxylic acids are removed from paper due to cleaning. As shown in Figure 3, peaks, at 4.5 and 6 min, attributable to oxalic and ascorbic acids (see Table S1), are reduced after PVAMB treatment, confirming the effectiveness of the proposed method. Importantly, no PVAMB residual was observed on the paper samples after treatment, because PVAMBs have a peak of around 2.5 min (Figure S2).

**Figure 4:**
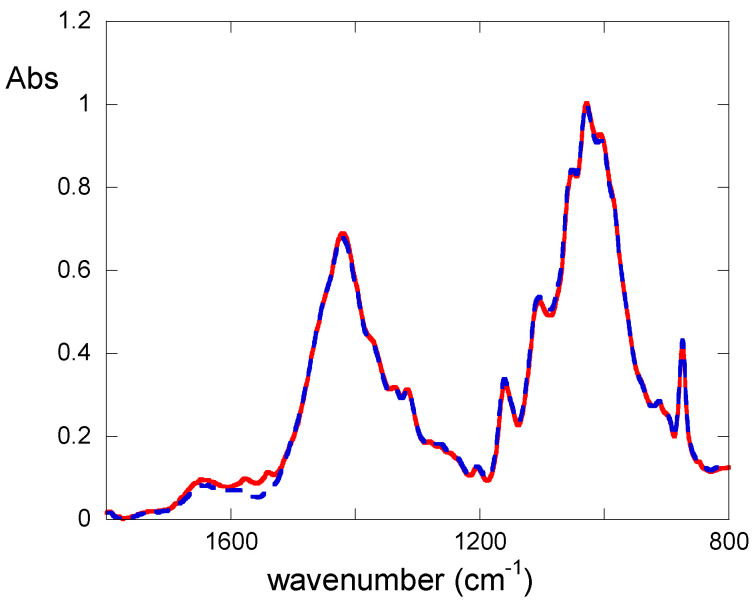
FTIR spectra of modern paper samples uncleaned (red continuous line) or treated using PVAMBs (blue dashed line).

Confirmation of PVAMB cleaning efficacy as well as (even if to a minor extent due to the lower detection limit of the method) of the absence of residues after the cleaning was obtained via FTIR experiments (Figure 4). FTIR spectra display the typical feature of cellulose moiety, i.e., a diagnostic band between 900 cm^−1^ and 1280 cm^−1^, attributable mainly to the bending modes of CCH, COC, and COH groups present in the glucose molecules [42], and of cellulose oxidative byproducts carrying carboxyl and carbonyl groups, which display absorption bands in the 1800–1500 cm^−1^ region [4]. The infrared spectra of the notebook samples show that the absorbance of the bands in the 1800–1500 cm^−1^ range decrease after cleaning, indicating the effectiveness of the removal of these byproducts with PVAMB treatment. Moreover, no additional peaks (as explained before, within the detection limit of the technique) attributable to PVAMBs (whose spectrum is reported in Figure S3) were present in the spectrum of the cleaned sample, thus suggesting the absence of PVAMB residues.

The efficiency of removal of chromophores (which are oxidized cellulose byproducts carrying conjugated carbonyl groups, [5,43]) was determined using color variation measurements. After cleaning, a chromatic variation of ΔE* = 2.4 ± 0.4 (too little to be observed by the naked eye, as evidenced in Figure 1) arising from an increase in brightness (ΔL* = 2.3 ± 0.2) and a decrease in the red (Δa* = −0.3 ± 0.1) and yellow (Δb* = −0.5 ± 0.1) tones occurred. This means that cleaning is able to remove oxidized cellulose byproducts responsible for the yellowing of paper samples.

### 2.2. Cleaning of Ancient Paper Samples

**Figure 5:**
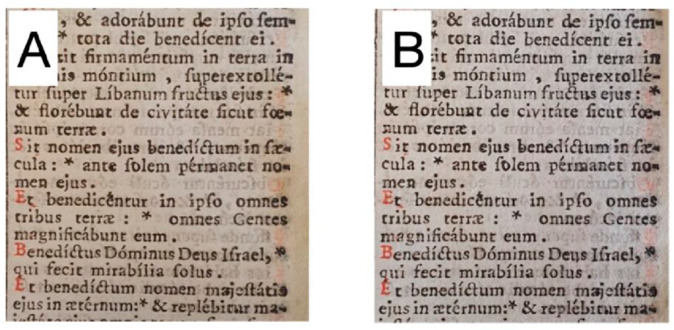
Ancient paper sample of *Breviarium* (see text) before (**A**) and after (**B**) cleaning with the proposed method.

Taking into consideration the great differences in terms of composition (and ageing) between modern and ancient paper samples, as well as manufacturing processes [2,16], the cleaning protocol used should be tuned to be compatible and effective on ancient paper samples. To this end, preliminarily, parameters used for PVAMBs stimulated using the US protocol were investigated on Whatman n°1 paper specimens (made up of pure cellulose paper, free of pollutants, fillers or glue). Experiments performed using the same cleaning protocol (as regards sonicator parameters) adopted for modern paper (consisting of a duty cycle of 100%, an intensity equal to 5 W/cm^2^, a frequency of 1 MHz and an application time of 2 min) provoked rips, probably because ancient paper is more delicate than modern paper. To this end, it was decided to test the lower intensity and duty cycle values of the sonicator. The most promising parameters included a duty cycle equal to 50% using an intensity of 2.5 W/cm^2^ while maintaining the same application time. The effectiveness of the proposed protocol was assessed using a multidisciplinary approach and is reported in the “Cleaning of ancient paper samples: protocol evaluation and compatibility tests on Whatman paper” paragraph in the Supplementary Materials (see also Figures S4–S7 and Table S2). Then, the proposed cleaning treatment was tested on naturally aged paper samples, which are samples from the *Breviarium*. As shown in Figure 5, cleaning does not induce visible paper damages and ink spreading, while also reducing paper yellowing.

**Figure 6:**
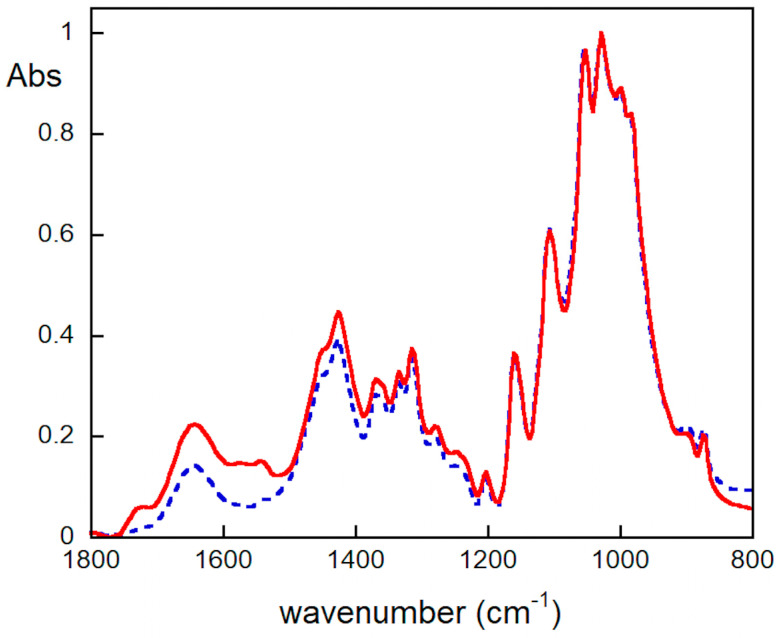
FTIR spectra of *Breviarium* paper sample before (red continuous line) and after (blue dotted line) cleaning using the proposed method.

As a quantitative measure, first of all, FTIR experiments on samples before and after cleaning were performed. As shown in Figure 6, treatment with PVAMBs provides a strong decrease in the absorbance of the FTIR bands in the 1750–1500 cm^–1^ range, indicating, as explained earlier, an effective removal of cellulose degradation byproducts. Furthermore, from this data, it is possible to obtain the oxidation index (OI), a parameter useful for establishing the degradation degree of the material under study. The OI value for the *Breviarium* paper sample after cleaning decreases from 0.47 ± 0.03 to 0.27 ± 0.03. This finding ascertains the improvement of the health state of paper thanks to PVAMBs treatment.

**Figure 7:**
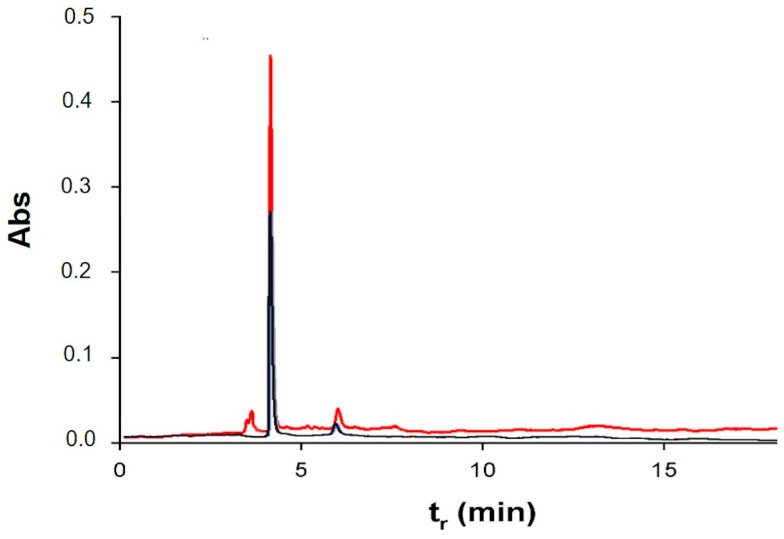
Chromatograms of aqueous extracts of *Breviarium* paper sample before (red) and after (blue) PVAMB cleaning.

The removal of acid molecules derived from cellulose deterioration was also proven by the rise in the pH value of paper after cleaning (pH values before and after treatments went from 5.9 ± 0.1 to 6.5 ± 0.1, respectively). More insight into the cleaning action of PVAMBs on paper was obtained via HPLC measurements of water extracts of *Breviarium* fragments (Figure 7). The use of PVAMBs leads to the removal of acids from the sample, as suggested by the decrease in the absorbance of peaks indicative of acid cellulose byproducts, these being oxalic acid and ascorbic acid (t_r_ = 4.5 min and 6 min, respectively). In addition, in this case, no peaks due to PVAMBs are present, indicating that no residues of the cleaning agents remain on the paper.

**Figure 8:**
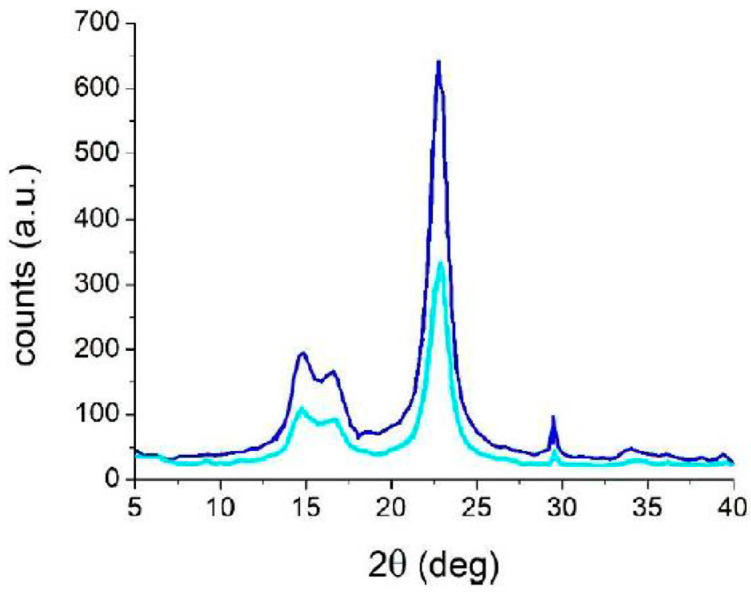
Diffraction pattern of Breviarium paper samples before (blue) and after treatment (light blue).

The improvement of the optical features of paper due to cleaning was assessed using colorimetric measurements. Indeed, a significant increase in brightness (ΔL* = 6.7) and a decrease in the red (Δa* = 1.4) and yellow (Δb* = 4.3) tones took place and an overall good chromatic variation (ΔE* = 8.1) on cleaned paper with respect to that of uncleaned paper was. This means that cleaning was able to remove oxidized cellulose byproducts responsible for paper sample yellowing. Finally, XRD analysis and tensile tests were performed in order to investigate the possibility of paper weakening induced by the PVAMB treatment of *Breviarium* paper (Figure 8). Additionally, in this case, as for modern paper, in the diffraction patterns of both treated and not treated *Breviarium* samples, in the 10–25° range, the peaks attributable to the (10$\overset{-}{1}$) plane and to the (00$\overset{-}{2}$) plane of the structure of cellulose I type β (PDF card no. 3–289) are present (Figure 8).

The calculated CI parameter (see above) for both samples, before and after the cleaning procedure, was found to be 0.9. This experimental evidence indicates that the proposed cleaning method has no effect on the crystallinity of cellulose. This result was confirmed using tensile tests. Stress values at the break values on samples were almost identical within the error before and after the cleaning procedure, being 5.4 ± 1.9 MPa and 5.3 ± 0.8, respectively. The strain values at the break values for the untreated and treated samples were also similar (being, in percentages, 1.4 ± 0.4 and 1.5 ± 0.3, respectively). Both analyses demonstrated that the proposed PVAMB and US treatment does not induce any mechanical damage on ancient paper.

## 3. Conclusions

The objective of this work was to enhance a cutting-edge “green” cleaning method applicable to modern and ancient paper samples in order to obtain a biocompatible, water-based tool for the simultaneous removal of hydrophilic (i.e., cellulose degradation byproducts) and hydrophobic (i.e., aged adhesive) substances from a sample in a short time. The suggested cleaning strategy is based on the use of an aqueous dispersion of poly (vinyl alcohol) microbubbles (PVAMBs) combined with 1 MHz ultrasounds. Due to their dimensions, of micrometer scale, they are able to enter paper pores, and thanks to the dynamics induced by US, they perform their cleaning action in just a few minutes. A protocol for the delicate removal of cellulose degradation byproducts, from modern notebook paper to naturally aged ancient paper, has been obtained using micro-invasive and non-invasive experimental techniques on paper samples before and after PVAMB cleaning. Th results obtained demonstrate the efficacy of the proposed cleaning method, which leads to an improvement in the readability of written paper samples, without affecting paper mechanical properties, causing ink spreading or leaving residues. This is the first attempt to assess this green biocompatible and rapid cleaning process as being generally usable for paper cleaning treatment. 

## 4. Materials and Methods

### 4.1. Reagents and Materials

Fully hydrolyzed PVA, NaIO_4_, HCl, fluorescein isothiocyanate (FITC), methanol, dimethyl sulfoxide (DMSO) and Whatman^TM^ n°1 filter paper samples were from Merck (Merck KGaA, Darmstadt, Germany). All reagents were of analytical grade and used without further purification. All water solutions were prepared using double-distilled water (Millipore, Billerica, MA, USA). Naturally aged paper samples (ancient paper) were from “*Breviarium Romanum ad usum fratum*” belonging to the XVIII century (grammage: 81 g/m^2^), from a private collection. The modern paper sample was from 1990s commercial, non-satinated notebook paper (grammage: 69 g/m^2^; mod. Clarissa Maxi, Blasetti, Pomezia, Italy).

### 4.2. Synthesis and Cleaning Procedures

#### 4.2.1. PVAMBs Synthesis and Characterization

The PVAMBs were prepared following a previously reported protocol [27,44]. Accordingly, 4 g of PVA was added to 200 mL of Milli-Q water and the suspension was heated at 80 °C while stirring until PVA solubilization occurred. Successively, about 0.4 g of sodium metaperiodate (NaIO_4_) was added, while stirring, to carry out the selective oxidation reaction of the vicinal hydroxyl groups and to induce the formation of aldehydic moieties in the PVA polymer so as to obtain telechelic PVA. The subsequent crosslinking reaction was then carried out, at room temperature for 2 h with vigorous stirring, at 8000 rpm (Ultra Turrax, IKA-Werke GmbH&Co. KG, Staufen, Germany) to form PVAMBs from the embedded air through agitation, while the telechelic PVA acted as a colloidal stabilizer. The so-formed PVAMBs were then washed from the unreacted reagents and solid residuals via flotation through a separatory funnel for several days, by using double-distilled water. To carry out a further purification of the PVAMB sample, any impurities or PVA residues were eliminated by centrifuging it many times in the suspension at 1000 rpm for 10 min; in this way, the supernatant containing the PVAMBs was completely isolated. The PVAMBs so-obtained were then kept stored in MilliQ water until further use. In order to determine the sample concentration in terms of number density (MBs/mL), 5 μL of the so-obtained solution was diluted in a ratio of 1:2 and put in a cell-counting chamber (Neubauer, Darmstadt, Germany), and the microbubbles were analyzed using an optical microscope (Nikon Inverted Microscope Eclipse Ti-E, Nikon, Newton, NJ, USA) with a 40× long distance objective (Nikon, Newton, NJ, USA) using the ImageJ freeware for the automated counting of the PVAMBs. The final concentration was about 2 × 10^8^ MBs/mL [35]. After preparation, the acoustic characterization of PVAMBs was also accomplished as measured using attenuation spectroscopy. The acoustic resonance, viscoelastic, and thickness features of the PVAMBs’ shells are comparable to the values reported in the literature [30]. To evaluate their mean diameter and size distribution, the PVAMBs’ shells were labelled with a fluorescent probe; in this case 50 μL of a solution of fluorescein isothiocyanate (FITC) in a concentration of 5 mg/mL in DMSO was added to 5 mL of the PVAMB starting solution. After 1 h of stirring in the dark, the solution was centrifuged at 1000 rpm for 10 min to remove the unreacted fluorophore. The FTIR spectrum of lyophilized PVAMBs, reported in Figure S8 [45,46], allowed an assessment of the presence of a PVA-based shell in the PVAMBs, as the characteristic bands of PVA at 1141 and 1090 cm^−1^ due to the C-O stretching modes of the polymer in the crystalline and amorphous parts were present [47]. With respect to the spectrum of fully hydrolyzed PVA, the FTIR profile of PVAMBs shows a decrease in the 1140 cm^−1^/1095 cm^−1^ band intensity ratio, suggesting a decrease in crystallinity as expected due the PVAMBs shell formulation process (i.e., polymer solubilization, oxidation and reticulation at the air/water interface; see above). Moreover, a shift in the maximum of the band at 1095 cm^−1^ due to the co-presence in the hydrogel and PVAMBs not only of the C-O of alcohol groups but also of acetal ones is found, confirming the presence of reticulation. Acoustic, viscoelastic, and shell thickness properties of PVAMBs have been already shown in the literature by authors [30,31,35]. Accordingly, PVAMBs’ resonance frequency (ω_0_) is found to be centered at about 11 MHz, as measured via acoustic attenuation spectroscopy [30]; their shear modulus and the shell thickness are, respectively, ~0.16 MPa and ~350 nm, as measured via attenuation spectroscopy and atomic force microscopy (AFM) [31,35]. It is interesting to note that, as already pointed out [35], PVAMBs’ morphological and viscoelastic properties still show no significant variations after more than a month, thus indicating the quite long-lasting stability of the system and, therefore the reproducibility of the treatment of the interface between paper and PVAMBs. The PVAMBs’ mean diameter was determined to be 3.60 ± 0.01 µm through a Gaussian fit being applied on confocal laser scanning microscopy results on the average distribution of PVAMBs (see below) [31]. Such a value was found to be coherent to the one determined through the dynamic light scattering experiments (see below), these being 3.50 ± 0.02 µm (Figure S9).

#### 4.2.2. PVA Hydrogel Synthesis and Characterization

PVA hydrogels were prepared in accordance with a procedure reported elsewhere [27,28]. The ratio of PVA and tel-PVA in the used hydrogels was 5% and 10%, respectively. To prepare tel-PVA, 2% (mol/mol of PVA repeating units) of solid NaIO_4_ was added to a PVA solution; then, the so-obtained solution was stirred at 60 °C, for 20 min, thus allowing the complete oxidation of the head-to-head PVA sequences. Successively after cooling to room temperature, an aqueous PVA solution at about 80 °C was added, and the solution was acidified at pH = 2.0 using HCl. The system was left for 24 h in the reaction vessel to allow the crosslinking of the polymer chains and gel formation. The gels were then repeatedly washed for several days using double-distilled water until the conductivity of water was about 1 μS, and no unreacted PVA traces were detected, as determined by performing ATR-FTIR analysis on dried residues of the used washing water. The FTIR spectrum of dried hydrogel is shown in Figure S8, in which bands at 1141 and 1090 cm^−1^ diagnostic of PVA are present. As for PVAMBs, in this case, compared to that of fully hydrolyzed PVA alone, a decrease in crystallinity (evidenced, as reported above, by the decrease in the band at 1140 cm^−1^, with respect to that at 1095 cm^−1^) and the formation of acetals (as suggested by a shift of the band at 1095 cm^−1^) were found. This result indicates crosslinking between PVA and tel-PVA, and in turn, gel formation.

The assessment of the formation of a hydrogel suitable for paper cleaning has been accomplished using rheology experiments. As already shown in the literature by authors [28], both the storage and the loss moduli, G’ and G’’, respectively, are constant and equal to 28,180 and 483, respectively, in a quite large range of applied deformations (0.1–10 rad/s) within a linear regime under a constant strain of 0.5%. It is important to note that G’ is about 58 times higher than G”, indicating that the gels have a solid-like behavior. Furthermore, the results obtained from the evaluation of the total water content inside the hydrogels’ structure showed the significant ability of the network’s material to retain water (97 ± 5), confirming the outcomes and characteristics already assessed in our previous work [29].

#### 4.2.3. Protocol Application

The proposed protocol consists of several steps. Firstly, is the application of a small amount of dispersed PVAMBs (the used volume is about 400 μL) on the selected paper area (about 2 × 2 cm^2^). Then, the working parameters of the sonicator were determined according to the desired protocol. Successively, the tip of the sonicator was applied to the sample and the cleaning treatment was exerted rotating the tip of the sonicator probe on it while a US pulse (Sonidel SP100 Sonoporator; Sonidel Limited, Dublin, Ireland) was applied (2 min unless specified). Finally, the removal of PVAMBs from the paper surface was ensured by dabbing the sample with a piece of chemical PVA gel for a few seconds. Temperature during the treatment was monitored using a tip thermometer, HI151 Checktemp 4, with a 0.1 °C accuracy (Hanna Instruments Inc., Woonsocket, RI, USA). The cleaning was performed at room temperature; the temperature did not exceed 35.0 °C during cleaning and dropped after the US was switched off.

### 4.3. Techniques

#### 4.3.1. ATR-FTIR Spectroscopy

FTIR spectra of paper samples were obtained with the mod. is50 Thermo Scientific instrument (Thermo Scientific Inc., Waltham, MA, USA) in attenuated total reflectance (ATR) mode using a single-reflection diamond ATR cell. Spectra were recorded in the wavenumber’s region from 4000 to 600 cm^−1^. Each spectrum was obtained by averaging over 32 scans with a resolution of 2 cm^−1^. Three experiments were performed in triplicate for each sample, yielding consistent and reproducible results. From the FTIR data it is possible to calculate the oxidation index (O.I.); that is, the ratio of the intensity of bands in the 1800–1664 cm^−1^ region with respect to that localized in the 1660–1500 cm^−1^ zone [4]. This parameter is useful for determining the health state of paper artworks. The bands attributable to carboxyl and carbonyl groups, which are formed at the cellulose final degradation stages, indeed, are localized at higher energies (1745–1710 cm^−1^) than are the bands attributable to the presence of cellulose oxidation intermediates (1660–1620 cm^−1^). Thus, as the O.I. values increased, the sample became increasingly degraded.

#### 4.3.2. X-ray Diffraction (XRD) Analysis

The structure and degree of crystallinity of the samples were characterized via X-ray diffraction (XRD) measurements performed using an XRD 3003 Seifert θ/2θ diffractometer (power system of 2200 W; Cu Kα anode target; beam dimension of 1 × 12 mm^2^; angular resolution of 0.001°). The XRD pattern was obtained using 40 kV tension, a 30 mA current, a 1 s/step acquisition time and a 0.02°/step angular scan. The crystallinity index (C.I.) of all samples was obtained by evaluating the difference in intensity between the two peaks assigned to cellulose as expressed in the following formula according to Segal and co-workers [48]:

CI = [(I_002_ – I_am_)/I_002_] × 100

where I_am_ is the intensity of the XRD signal due to the “amorphous” part of cellulose (i.e., the (101) and (10$\overset{-}{1}$) reflections), and I_002_ is the intensity of the crystalline part.

#### 4.3.3. Tensile Tests

Tensile test, one of the most common mechanical testing techniques was performed on paper specimens with dimensions 200 × 15 mm^2^, using the universal testing machine (Lloyd LRX) equipped with a load cell of 50 N. A gauge length of 80 mm, a crosshead speed of 5 mm/min, and a preload of 0.2 N were set following the UNI EN ISO 1924-2:2009 standard. All experiments were performed tenfold, yielding consistent and reproducible results.

#### 4.3.4. High-Performance Liquid Chromatography (HPLC) Analysis and pH Measurements

HPLC analysis was performed using a THERMOQUEST instrument (Shimadzu, Kyoto, Japan), equipped with two pumps and an ultraviolet/visible (U*V*/*v*is) detector, LCGA SPD-10A (Shimadzu, Kyoto, Japan), equipped with a 4.6 x 250mm chromatographic column, HPLC Pinnacle II C18, which porosity is 5 μm, (RESTEK Corporation, Centre, PA, USA). Chromatographic analysis for the determination of organic acids arising from the process of paper degradation (compounds characterized by low-molecular-weight and hydrophilic behavior) was performed on water extracts obtained by soaking 1 cm^2^ of every paper sample with 1.5 mL of distilled water, stirring using the rotating wheel (Dynal Biotech-Life Technologies Italia Fil., Monza, Italy) overnight at room temperature. The analysis of paper samples was performed before and after the cleaning procedure. This analysis was carried out in an isocratic condition using a 25 mM phosphate buffer at pH 2.4 and 10% (*v*/*v*) methanol as a mobile phase. The flow rate was 0.8 mL/min, with a loop of 200 μL and the detection wavelength being λ = 230 nm. Measurements of pH were carried out on the paper surface using an Crison pH meter, mod. Basic-20(Crison Instruments s.a. Alella, Barcelona, Spain), equipped with a flat-tipped electrode (mod. HI1413B; Hanna Instruments Inc., Woonsocket, RI, USA) At least three measurements were performed for each sample.

#### 4.3.5. Colorimetry

Colorimetry measurements concerning the optical quality of paper were performed using Eoptis Digital Handheld Colorimeter (mod. CLM-194; Eoptis srl, Trento, Italy). Coordinates in the CIELAB color space (L* = brightness, a* = red/green color component, b* = blue/yellow color component) were obtained using a D65 illuminant and a 10° observer. For each sample, at least three measurements were performed.

#### 4.3.6. Optical Microscopy

Phase-contrast and confocal microscopy were used to analyze the number density and size distribution of the PVAMBs, by using Inverted Microscope Eclipse Ti-E (Nikon, Newton, NJ, USA), equipped with 20× and 40× long-distance objectives (Nikon). To determine the size distribution of the PVAMBs, confocal laser scanning microscopy (CLSM) was used, with Nikon Inverted Microscope Eclipse Ti-E coupled with a Spectra Physics Ar ion laser as a light source (λ = 488 nm), and a Plan Apo 60× oil immersion objective with NA = 1.4. The instrument was equipped with the software Elements AR Analysis 4.30.02 64-bit.

#### 4.3.7. Dynamic Light Scattering (DLS) Measurements

Dynamic light scattering (DLS) experiments were conducted to assess the average diameter and the size distribution of the PVAMBs, by using DLS BI-9000AT (Brookhaven Instr. Corp., New York, NY, USA), equipped with a BI-200SM goniometer and a solid-state laser (λ_em_ = 532 nm). These experiments were carried out at room temperature, by diluting a sample of dispersed PVAMBs in a ratio if 1:4, and placing the sample in an appropriate cuvette. The correlation function of the obtained scattered intensity was analyzed using the algorithm CONTIN included in the software.

## Data Availability

Data will be made available upon request.

## Supplementary Materials

The following supporting information can be downloaded at https://www.mdpi.com/article/10.3390/gels9070509/s1. Figure S1. Examples of modern paper samples uncleaned (A), treated with PVAMBs and US (B) or untreated (b); Figure S2. Chromatograms of PVAMBs in water; Figure S3. FTIR spectra of notebook paper sample after treatment (red) and freeze-dried PVAMBs (blue). A paragraph titled: “economic feasibility evaluation of PVAMBs system”. A paragraph titled: “Cleaning of ancient paper samples: protocol evaluation and compatibility tests on Whatman paper” containing Figures S4–S7 and Table S2. Figure S4. Phase-contrast images of Whatman filter paper sample before (A) and after (B) protocol application (bar scale: 15 μm; magnification: 40X); Figure S5. Diffraction pattern of Whatman filter paper sample before (blue) and after (light blue) protocol application; Figure S6. HPLC chromatograms from water extracts of Whatman filter paper sample before (blue) and after (red) PVAMBs application; Figure S7. FTIR spectra of Whatman filter paper sample before (red) and after (blue) protocol application; Table S2. pH and colorimetric values obtained on Whatman paper before and after different cleaning approaches. Figure S8. FTIR spectra of (A, black line): fully hydrolyzed PVA (in the powder form); (B red line): lyophilized PVAMBs; (C, blue line): dried PVA hydrogel. Figure S9. (a) Confocal laser scanning microscope image of FITC-labeled PVA MBs (60X oil immersion obj, small pinhole and 6.10 gain); (b) histogram and relative Gaussian fit of the mean diameter distribution of the MBs obtained through CLSM; (c) histogram showing the mean diameter of the PVA MBs, obtained by applying the CONTIN algorithm on DLS data; A paragraph concerning the economic feasibility evaluation of PVAMBs system [3,5,39,40,43,45,46,49].
