# Supplementary material for: Ultrasound-Stimulated PVA Microbubbles as a Green and Handy Tool for the Cleaning of Cellulose-Based Materials
Source: Gels. 2023 Jun 23;9(7):509. doi: 10.3390/gels9070509 (PMC10379172; doi:10.3390/gels9070509)
Supplement: Supplementary file 1 [file gels-09-00509-s001.zip › gels-2425782-supplementary final.pdf]

gels

Supplementary Materials

Ultrasound-Stimulated PVA Microbubbles as a Green and Handy Tool for the Cleaning of Cellulose-Based Materials

Leonardo Severini [1], Alessia D'Andrea [1], Martina Redi [1], Sultan B. Dabagov [2,3,4], Valeria Guglielmotti [2], Dariush Hampai [2], Laura Micheli [1], Rocco Cancelliere [1], Fabio Domenici [1,*], Claudia Mazzuca [1,*], Gaio Paradossi [1] and Antonio Palleschi [1]

[1] Department of Chemical Science and Technologies, University of Rome "Tor Vergata", Via Della Ricerca Scientifica 1, 00133 Rome, Italy; leonardo.severini@uniroma2.it (L.S.); alessia.dandrea@alumni.uniroma2.eu (A.D.); martina.redi@uniroma2.it (M.R.); laura.micheli@uniroma2.it (L.M.); rocco.cancelliere@uniroma2.it (R.C.); gaio.paradossi@uniroma2.it (G.P.); antonio.palleschi@uniroma2.it (A.P.)

[2] INFN-LNF, XLab Frascati Via Enrico Fermi 54, 00044 Frascati, Rome, Italy; sultan.dabagov@lnf.infn.it (S.B.D.); valeria.guglielmotti@lnf.infn.it (V.G.); dariush.hampai@lnf.infn.it (D.H.)

[3] RAS P.N. Lebedev Physical Institute, Leninsky pr 53, 119991 Moscow, Russia

[4] National Research Nuclear University MEPhI, Kashirskoe Sh. 31, 115409 Moscow, Russia

* Correspondence: fabio.domenici@uniroma2.it (F.D.); claudia.mazzuca@uniroma2.it (C.M.)

Introduction

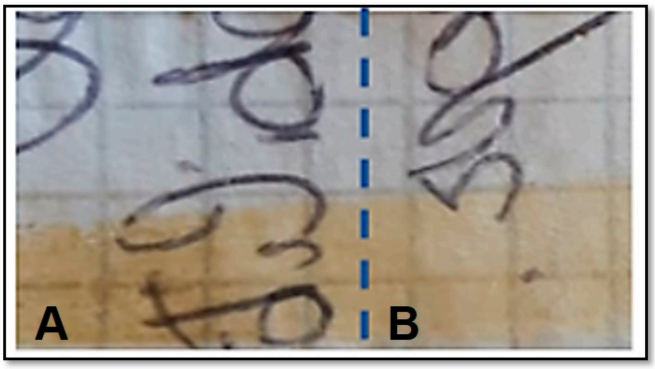

(a)

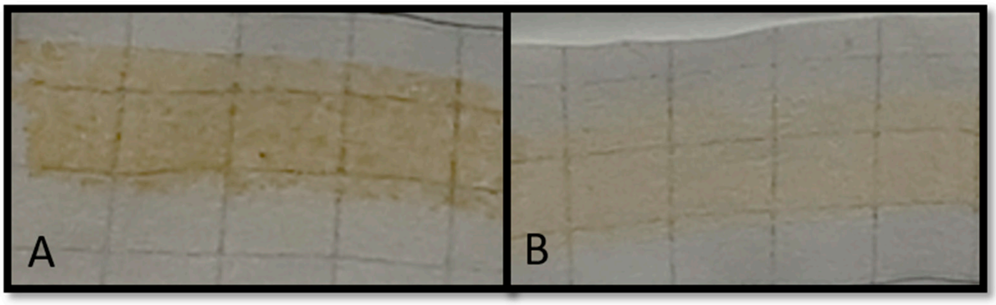

(b)

Figure S1. Two examples (named **a** and **b**) of modern paper samples (A): uncleaned; (B): treated with PVAMBs and USEconomic feasibility evaluation of PVAMBs system.

Localized wet cleaning treatments sometimes need to be performed on sensitive paper artifacts, and require flexible, compatible, easy-to-use systems. Nowadays there are several challenges for restorers and a balance must be found between efficacy, compatibility, and processing times. In this context, the economic feasibility of the treatment also represents a crucial matter of debate since such treatments often have to be

performed on large surfaces (e.g., over artwork of large dimensions or several pages of a book).

In this complicated scenario the PVAMBs based cleaning system presented in this study, is a good alternative in terms of stability, reproducibility, and efficacy as much as in terms of economic affordability, compared with the more common alternatives above reported.

The non-toxic materials (see *"Materials and Methods"* section in the main manuscript) needed for both the synthesis of PVAMBs of PVA hydrogel are quite commonly available and can be found at a range of affordable prices (~60—170 €). Given the relatively small amount required of reagents, approximately 25g of fully hydrolyzed polymer and of the oxidant agent is enough to perform about six batches each of 50 mL of PVAMBs solution.

Furthermore, it has been estimated that, using a single batch of PVAMBs, it should be possible to clean about four-five pages with dimensions of about 15 × 8 cm^2. It should be noted, moreover, that the US instrument, required for US stimulation of PVAMBs, is easily available in the market and costs about 500 €.

Furthermore, the affordable initial costs are amortized by the robustness of the instrument performance, which can be guaranteed for at least 10 years and does not require maintenance or replacement of accessories.

Results and Discussion

Table S1. Retention time of organic acids used as standards for HPLC chromatograms of aqueous extracts of paper samples.

Acid	Retention Time (t_r; minutes)
Oxalic acid	4.7
Ascorbic acid	6.2
Lactic acid	6.8
Citric acid	7.0
Succinic acid	7.6

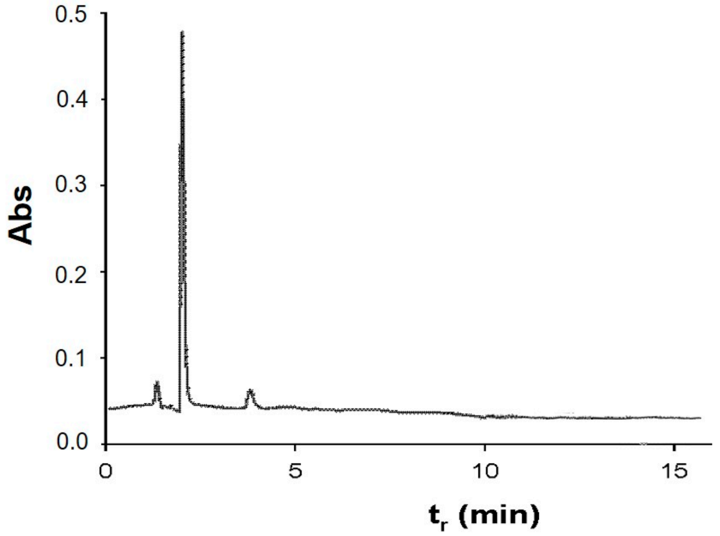

Figure S2. Chromatograms of PVAMBs in water.

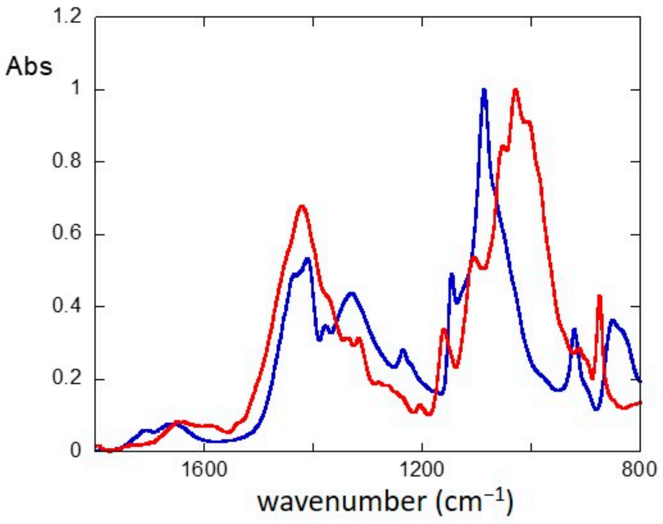

Figure S3. FTIR spectra of notebook paper sample after treatment (red) and freeze-dried PVAMBs (blue).

Cleaning of ancient paper samples: protocol evaluation and compatibility tests on Whatman paper

Optical microscopy images (Figure S4) show that the morphology of the cellulose fibers does not change before and after the cleaning with PVAMBs using the proposed protocol (a duty cycle = 50%; intensity = 2.5 W/cm^2) demonstrating that no visible damages have been produced.

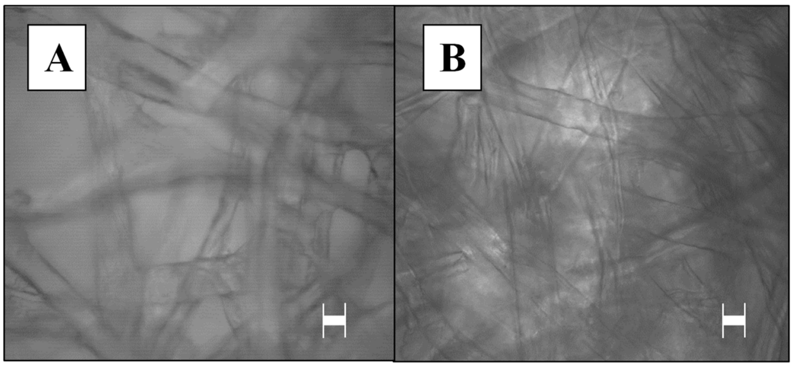

Figure S4. Phase-contrast images of Whatman filter paper sample before (**A**) and after (**B**) protocol application (bar scale: 15 μm; magnification: 40X).

This result is confirmed by XRD data. Both X-ray diffraction patterns of untreated and treated Whatman paper (Figure S5) indeed, show peaks in the 10—25° range of the diffraction pattern. The first is assignable to the $(10\bar{1})$ plane relative to the structure of cellulose I type β (PDF card no. 3—289); the second most intense peak is assigned to the $(00\bar{2})$ plane, and represents the crystalline part of the cellulose [39,40].

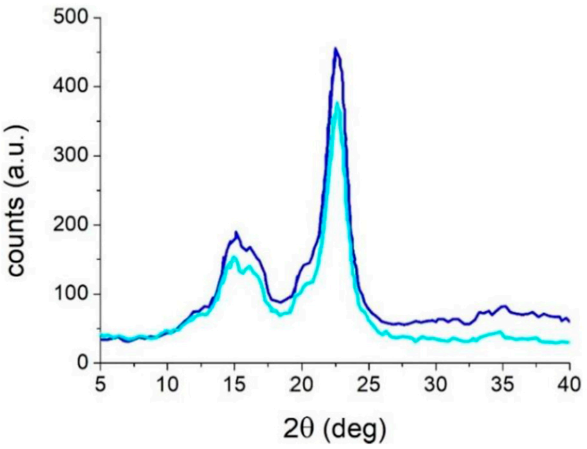

Figure S5. Diffraction pattern of Whatman filter paper sample before (blue) and after (light blue) protocol application.

Importantly, the crystallinity index (CI) values of the paper samples, before and after the cleaning procedure are comparable and equal to 0.8, highlighting that the cleaning protocol did not damage the sample.

Regarding the cleaning efficacy, pH and colorimetric measurements were performed before and after treatment with PVAMBs. As explained in the main text, degradation indeed causes a decrease in pH due to formation of acid molecules [3,49], and a worsening of optical quality (yellowing) due to the formation of cellulose byproducts carrying carbonyl groups [5,43]. As shown in Table S2, because of cleaning there is a slight increase in both pH and ΔE* values, thus indicating that the treatment with PVAMBs is able to remove degradation products, that are eventually present even in well-preserved reference paper.

Table S2. pH and colorimetric values variation obtained on Whatman paper before and after different cleaning approaches. (L* = brightness, a* = red/green color component, b* = blue/yellow color component, E* = whole chromatic value)

Treatment	pH	ΔL*	Δa*	Δb*	ΔE*
Untreated	5.2 ± 0.2				
PVAMBs (2 min)	5.9 ± 0.2	0.07± 0.01	0.02 ± 0.01	−0.18 ± 0.01	0.19 ± 0.01

The removal of impurities or organic acids as cellulose degradation products (i.e., oxalic, and ascorbic acids that are retention peaks at 4.5 and 6 min, respectively) has been confirmed by HPLC measurements. As reported in Figure S6, indeed, the chromatographic peaks of the sample obtained from water extraction of paper cleaned using PVAMBs are less intense than that relative to uncleaned ones, thus confirming that acids are removed from Whatman specimens by PVAMBs. Furthermore, importantly, no additional peaks attributable to PVAMBs residues after the treatment, (see Figure S3) are present.

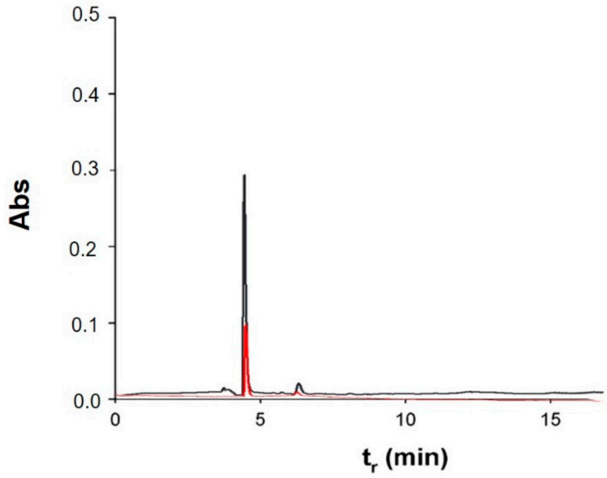

Figure S6. HPLC chromatograms from water extracts of Whatman filter paper sample before (blue) and after (red) PVAMBs application.

Finally, the absence of PVAMBs residues and the lack of changes in the crystallinity has been supported by FTIR experiments (Figure S7). The spectra of samples before and after cleaning with PVAMBs are superimposable, suggesting that the treatment does not leave detectable PVA residues on the paper samples. Furthermore, no alteration of the crystallinity of the material occurs, by ultrasound action, as demonstrated by no intensity changes of the band at 905 cm^{-1} band, as well as of the intensities of bands centered at 1427cm^{-1} and 898 cm^{-1} whose ratio value (1.4, before and after treatment) is related to the crystallinity of samples [45,46].

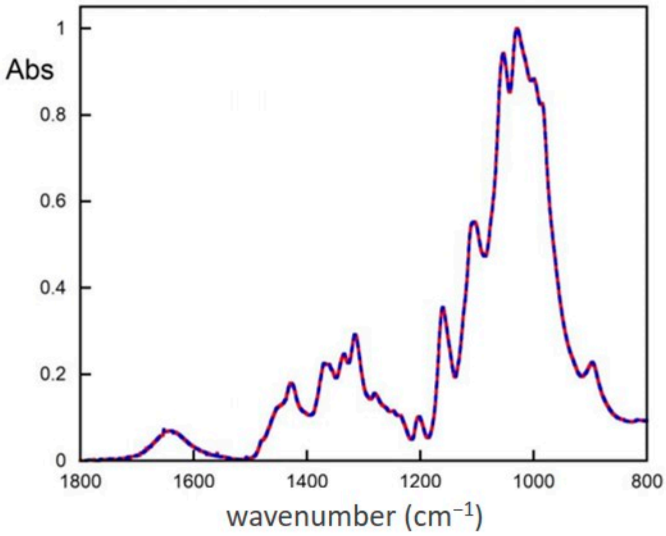

Figure S7. FTIR spectra of Whatman filter paper sample before (red) and after (blue) protocol application.

Materials and Methods

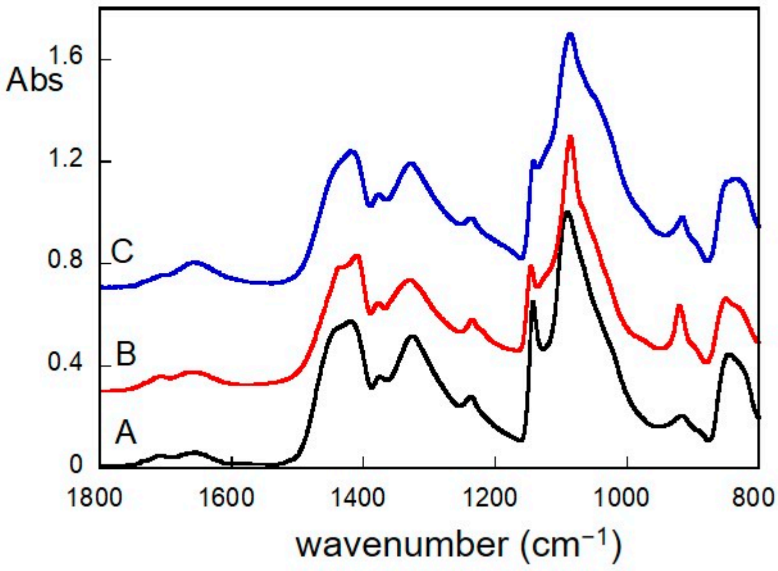

Figure S8. FTIR spectra of (A, black line): fully hydrolyzed PVA (in the powder form); (B red line): lyophilized PVAMBs; (C, blue line): dried PVA hydrogel.

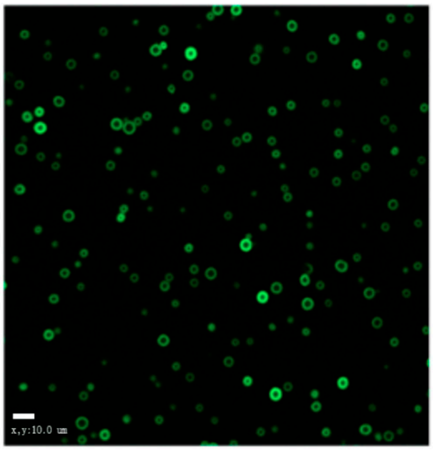

(**a**)

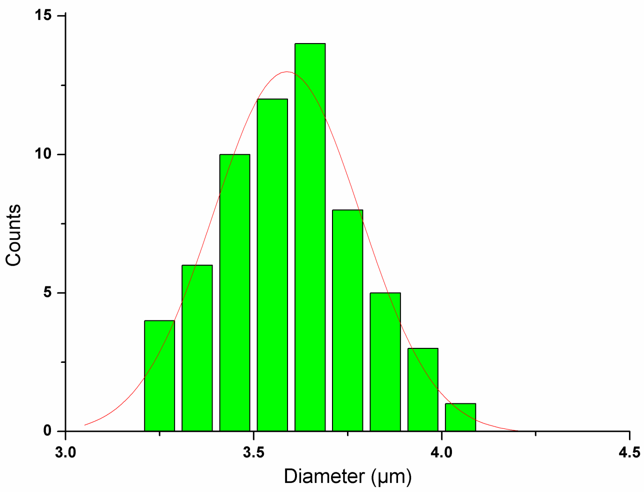

(**b**)

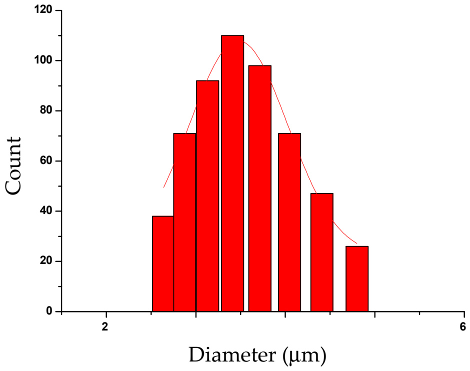

(**c**)

Figure S9. (**a**) Confocal laser scanning microscope image of FITC-labeled PVA MBs (60X oil immersion obj, small pinhole and 6.10 gain); (**b**) histogram and relative Gaussian fit of the mean diameter distribution of the MBs obtained through CLSM; (**c**) histogram showing the mean diameter of the PVA MBs, obtained by applying the CONTIN algorithm on DLS data.
